# Intraventricular infusion of quinolinic acid impairs spatial learning and memory in young rats: a novel mechanism of lead-induced neurotoxicity

**DOI:** 10.1186/s12974-018-1306-2

**Published:** 2018-09-14

**Authors:** Abdur Rahman, Muddanna S. Rao, Khalid M. Khan

**Affiliations:** 1 Department of Food Science and Nutrition, College of Life Sciences, Kuwait University, Kuwait City, Kuwait; 2 Department of Anatomy, Faculty of Medicine, Kuwait University, Kuwait City, Kuwait

**Keywords:** Quinolinic acid, Spatial learning, Memory, Lead, Neurotoxicity

## Abstract

**Background:**

Lead (Pb), a heavy metal, and quinolinic acid (QA), a metabolite of the kynurenine pathway of tryptophan metabolism, are known neurotoxicants. Both Pb and QA impair spatial learning and memory. Pb activates astrocytes and microglia, which in turn induce the synthesis of QA. We hypothesized increased QA production in response to Pb exposure as a novel mechanism of Pb-neurotoxicity.

**Methods:**

Two experimental paradigms were used. In experiment one, Wistar rat pups were exposed to Pb via their dams’ drinking water from postnatal day 1 to 21. Control group was given regular water. In the second protocol, QA (9 mM) or normal saline (as Vehicle Control) was infused into right lateral ventricle of 21-day old rats for 7 days using osmotic pumps. Learning and memory were assessed by Morris water maze test on postnatal day 30 or 45 in both Pb- and QA-exposed rats. QA levels in the Pb exposed rats were measured in blood by ELISA and in the brain by immunohistochemistry on postnatal days 45 and 60. Expression of various molecules involved in learning and memory was analyzed by Western blot. Means of control and experimental groups were compared with two-way repeated measure ANOVA (learning) and *t* test (all other variables).

**Results:**

Pb exposure increased QA level in the blood (by ~ 58%) and increased (*p* < 0.05) the number of QA-immunoreactive cells in the cortex, and CA1, CA3 and dentate gyrus regions of the hippocampus, compared to control rats. In separate experiments, QA infusion impaired learning and short-term memory similar to Pb. PSD-95, PP1, and PP2A were decreased (*p* < 0.05) in the QA-infused rats, whereas tau phosphorylation was increased, compared to vehicle infused rats.

**Conclusion:**

Putting together the results of the two experimental paradigms, we propose that increased QA production in response to Pb exposure is a novel mechanism of Pb-induced neurotoxicity.

## Background

Lead (Pb) is a well-known neurotoxicant [1, 2] that is still abundantly present in our environment despite the significant efforts to decrease its use and distribution in the environment [3–5]. Pb targets multiple organ systems such as the nervous, hematopoietic, immune, renal, reproductive, endocrine, and skeletal. The central nervous system (CNS) is most sensitive to Pb toxicity, particularly during early development. In recent years, the role of immune system in Pb-induced neurotoxicity has emerged, and several inflammatory mediators, including cytokines and enzymes involved in inflammatory process, are known to play a significant role in neurotoxicity and neurodegeneration. In particular, Pb-induced astroglyosis and microgliosis have been shown to be involved in neurotoxictiy [6, 7]. Compared to other systems, CNS is affected by doses of Pb as low as 7 μg/dL [2]. Based on the evidence that Pb exposure at levels below the safety limit of 10 μg/dL causes neurological alteration in children [2, 8], the US Centers for Disease Control and Prevention (CDC) recently revised the blood Pb level from 10 μg/dL, which was set in 1991, to 5 μg/dL as the reference level at which CDC recommends the initiation of public health actions [9]. The mechanism(s) of Pb-induced neurotoxicity is thoroughly investigated and several biochemical targets and pathways involved in neurotoxicity have been identified. However, the mechanism by which these biochemical abnormalities lead to impairment of learning and memory is poorly understood at best.

Quinolinic acid (QA) is a metabolite of the kynurenine pathway (KP) of tryptophan metabolism and is a known excitotoxic compound acting through the activation of the *N*-methyl-d-aspartic acid receptor (NMDAR) [8]. Approximately 95% of tryptophan is metabolized by the KP [9]. QA is toxic to oligodendrocytes [10] and neurons [13, 14]. QA toxicity affects neurons located mainly in the hippocampus, striatum, and neocortex [15]. In pathological concentration, QA promotes apoptosis in oligodendrocytes, neurons, and astrocytes [16–18]. Increased levels of QA are involved in neurodegenerative and neurological disorders such as Alzheimer’s disease, Huntington’s disease, amyotrophic lateral sclerosis, AIDS-dementia, cerebral malaria, depression, and schizophrenia [15, 19–22]. The mechanisms by which QA exerts its neurotoxic effects is through provoking enhanced intracellular calcium through over activation of NMDAR, augmented levels of extracellular glutamate, increased reactive oxygen species and reactive nitrogen species formation, decreased activity and expression of antioxidant systems, oxidative stress, stimulated protease activity, and cell death [20, 23–25]. QA-induced neurotoxicity also involves destabilization of the cytoskeleton [22] and energy depletion [15].

The major and rate-limiting enzyme of KP, indoleamine-2,3-dioxygenase-I, (IDO-I), is expressed in astrocytes, microglia, and neurons. The expression of IDO-I is increased by inflammatory mediators and cytokines such as amyloid peptides, LPS, IL-1β, TNF-α, and IFN-γ [11, 26, 27]. The other major enzyme of the KP that converts kynurenine (the first stable metabolite of KP) into QA is kynurenine 3-monooxygenase, which is also abundantly expressed in microglia, macrophages, and monocytes. The expression of this enzyme is also upregulated by pro-inflammatory mediators [28]. Thus, a pro-inflammatory environment highly favors the generation of QA in the brain. In addition to the local activation of the KP and synthesis of QA in the CNS, kynurenine produced systemically can cross the blood brain barrier and can be converted into QA within the CNS [29].

Both Pb and QA share several features of neurotoxicity. For example, in rats, both Pb [30] and QA [31–33] impair learning and memory. Similarly, both Pb and QA induce tau hyperphosphorylation [22, 34]. Tau hyperphosphorylation is associated with memory loss in Alzheimer’s disease and other dementias like FTD-17. In addition, QA is produced in response to oxidative stress and is also a pro-oxidant and induces oxidative stress [11, 23, 25–28]. Pb is known to be involved in oxidative stress. One of the reported mechanisms of QA neurotoxicity is increased accumulation of glutamate at the synapse by increasing its release from neurons and inhibiting its uptake by astrocytes [15]. Similar to QA, Pb is also known to increase spontaneous release of glutamate and GABA from the presynaptic terminal of rat hippocampal neurons [35].

In this study, we investigated the role of QA in Pb-induced impairment of learning and memory. We hypothesize that Pb, being a prooxidant, increases the brain levels of QA, which subsequently results in neurotoxicity and impairment of learning and memory. The specific aims were investigating the effect of the following: (1) Pb exposure on QA level in blood and QA immunoreactivity in different regions of the brain; (2) Pb exposure during lactation on spatial learning and memory; (3) intraventricular infusion of QA on spatial learning and memory; and (4) intraventricular infusion of QA on the expression of various molecules involved in learning and memory. We report here that Pb exposure increased QA level in blood and QA immunoreactivity in the brain of rats and that QA infusion in the brain produced behavioral and biochemical changes that are largely similar to that produced by Pb exposure. These results support the hypothesis that increased QA production in response to Pb exposure is involved in learning and memory impairment.

## Methods

### Animals

Wistar rats were provided by the Animal Resources Center, Faculty of Medicine, Kuwait University. The animals were housed at constant temperature (21 ± 2 °C) and relative humidity (50 ± 10%) with a 12-h light/dark cycle (0700–1900 h). The animal maintenance and exposure were according to the protocol approved by the Animal Care and Use Committee of Kuwait University and the experimental protocol followed the *ARRIVE* guidelines for the care and use of laboratory animals.

### Lead exposure protocol

At birth, pups were culled to 10 per liter and exposed to Pb via their dams’ drinking water (0.2% Pb acetate in deionized water) from postnatal day (PND) 1 to 21. Control group with similar number of pups was given deionized water. From PND21, both the groups (Pb-exposed and control) were given tap water until the termination of the study. In our previous experiments, similar exposure protocol produced a blood Pb level of 8.3 **±** 4.3 μg/dL in pups at PND21 [36]. Rats from both groups were subjected to Morris water maze (MWM) test from PND30 and from PND45. Short-term memory (STM) was assessed by probe test (memory retention test) which was done 48 h after the last learning session. Long-term memory (LTM) was assessed by probe test on PND45 or PND60. After the LTM retention test (PND45 and PND60, respectively), animals were euthanized with CO_2_. Thoracic cavities were opened, and blood was drawn from the right ventricle for measuring QA by ELISA.

### ELISA for quinolinic acid

Blood was centrifuged at 2000×*g* for 15 min. Serum was transferred to Eppendorf tubes and stored at − 80 °C until analysis. The ELISA for QA was conducted in serum by using QA ELISA kit (Aviva Systems Biology Corporation, San Diego, CA, USA; Cat. No. OKCD02284). Standard and the samples (50 μl) were added to the wells pre-coated with specific antibody in a 96-well plate, and the assay was conducted as per the manufacturer’s instructions. The results were calculated by fitting the optical density into a four-parametric logistic curve.

### Intraventricular infusion of quinolinic acid

Wistar rat pups (21-day old) were anesthetized with a mixture of ketamine (40 mg/kg) and xylocaine (5 mg/kg) and fixed in a stereotaxic frame. The coordinates were as follows: anteroposterior—3 mm behind bregma; lateral—3.6 mm from midline; and depth—3.8 mm from skull surface. Two holes were made, one for a steel cannula aimed at the right lateral ventricle and the other for the fixing screw. An Alzet Brain Infusion Kit (cannula and tubing) was used with Alzet 1007D osmotic minipump (Durect Corporation, CA, USA). QA (9 mM) prepared in sterile saline was infused in the right lateral ventricle for 7 days. Assembling and filling the osmatic minipump were done in a sterile culture hood. Filled osmotic minipumps were incubated in saline water bath maintained at 37 °C for 12 h to stabilize the pump and to check the flow. Rat pups infused with the same volume of sterile normal saline served as vehicle control (VC) group. Osmotic pumps were removed on 8th post implantation day. Spatial learning and memory was assessed by MWM test starting on PND30 and PND45 (two separate groups).

### Spatial learning and memory testing

Pb-exposed and QA-infused rats, along with their respective controls, were subjected to the MWM test at PND30 (control, *n* = 10; Pb-exposed, *n* = 14; vehicle control, *n* = 7; QA infused, *n* = 8) and a separate group at PND45 (control, *n* = 9; Pb-exposed, *n* = 13; vehicle control, *n* = 10; QA infused, *n* = 11). The water maze apparatus consisted of a water tank of 2.0 m in diameter, divided into four virtual quadrants. A circular platform was submerged in one of the quadrant (target/platform quadrant). The rats were trained in the water maze in six sessions on four consecutive days (one session on the first and last day and two sessions on the 2nd and 3rd day). Each session consisted of four trials; each trial was of 120-s duration. Inter-trial interval was 60 s. In each trial, time taken to reach the hidden platform (escape latency) was measured and analyzed using EzVideo™ 5.70 Digital Video Tracking system (Accuscan Instruments, Inc., Columbus, OH, USA). Two days and 10 days after the last learning session, rats were subjected to memory retention test (probe test), for short-term (STM) and long-term memory (LTM), respectively. Each probe test session was of 30-s duration. Data on several parameters, i.e., platform quadrant (zone) time, platform quadrant entry latency, and distance traveled in the target/platform quadrant, were measured and analyzed using EzVideoTM 5.70 Digital Video Tracking system.

### Western blotting

At the end of the experiment (PND45 and PND60, respectively for the two groups), animals were euthanized with CO_2_ and then decapitated. Skull cap was removed, and the brain along with the skull base was bisected. Cerebral hemispheres were placed in pre-weighed Eppendorf tubes and were snap-frozen in liquid nitrogen and stored at − 80 °C till analyses. Cerebral hemispheres were homogenized in five volumes of RIPA buffer (50 mM Tris, pH 7.4, 150 mM NaCl, 1% NP-40, 5 mM EDTA, 0.5% sodium deoxycholate, 0.1% SDS, 50 nM NaF, 1 mM sodium vanadate and protease inhibitor cocktail (Roche Diagnostic, Castle Hill, NSW, Australia) (PND30: vehicle control, *n* = 6; QA infused, *n* = 6) (PND45: vehicle control, *n* = 6; QA infused, *n* = 6). Protein in each sample was determined by the Bradford method and the homogenates were kept at − 80 °C till used. Protein (50 μg) from each sample was resolved on a 10% bis-tris SDS-PAGE (NuPAGE, Invitrogen, Carlsbad CA, USA) and transferred onto PVDF membrane. The membrane was blocked with 5% non-fat dry milk in TBS-Tween-20 for 1 h and incubated with specific antibodies (Table 1) overnight at 4 °C at a dilution of 1:500 or 1:1000 (Table 1). The membrane was rinsed with TBS-Tween-20 six times (10 min each). The membrane was then incubated with the HRP-conjugated appropriate secondary antibody (Sigma-Aldrich, St. Louis, MO, USA) for 2 h at room temperature, washed as before and developed with the ECL kit (Thermo Scientific). Actin was used as a loading control. After developing the radiographic film, protein bands were quantified by Syngene Genetool software.

**Table 1:** Details of primary antibodies used for Western blot

Antibody	Antigen/epitope	Source/reference
PP1 (E-9)*^, #^	PP1 Catalytic subunit	Santa Cruz Biotech
Anti-PP2A (Clone 1D6)*^, #^	PP2A Catalytic subunit	Merck Millipore
Anti-Synaptophysin (Clone SVP-38)*^, #^	Synaptophysin	Sigma-Aldrich
CREB (48H2)*^, #^	Total CREB-1 protein	Cell Signaling
Phospho-CREB (Ser133) (87G3)*^, #^	Ser 133-phosphorylated CREB	Cell Signaling
AT180*^, ##^	Pt231	Invitrogen
Anti-PSD95*^, ##^	Rat PSD95	Sigma-Aldrich
Anti-NR1*^, ##^	Amino acids 918–938 with N-terminally added lysine of the NR1 subunit of NMDAR	Sigma-Aldrich
Anti-phospho-NR1 (Ser897)**^, ##^	Phospho-Ser897 of human NMDA Receptor	Cell signaling
Anti-phospho-NR2B (Ser1303)**^, ##^	CKLRRQH(Ps)YDTFVD of NR2B	Merck Millipore
Anti-NR2B**^, ##^	1437–1456 of mature mouse NR2B	Merck Millipore
Tau Antibody (A-10)*^, #^	Total tau	Santa Cruz Biotech

*Monoclonal, **Polyclonal, ^#^1:500 dilution, ^##^1:1000 dilution

### Tissue processing for immunohistochemistry

At the end of probe test (PND45 for the PND30 group and PND60 for the PND45 group), rats were euthanized with CO_2_ and transcardially perfused with the fixative consisting of 4% paraformaldehyde and 0.1% glutaraldehyde in 100 mM phosphate buffer, pH 7.4 at 4 °C (PND45: control, *n* = 4; Pb-exposed, *n* = 4; PND60: control, *n* = 4; Pb-exposed, *n* = 4). Brains were transferred to cold fixative and kept in the fixative for 4–6 h at 4 °C. Tissues were washed overnight in cold phosphate buffer, dehydrated in graded ethanol, cleared in xylene, and embedded in paraffin. Sections were cut at a thickness of 6 μm on rotary microtome. Sections were deparaffinized, rehydrated, and washed with 10 mM phosphate buffered saline (PBS), pH 7.4 at room temperature. The sections were quenched for endogenous peroxidise activity and free aldehyde groups with 3% H_2_O_2_ in water and 50 mM glycine in PBS, respectively. The sections were then sequentially incubated in primary antibody (rabbit anti-quinolinic antibody, Cat. No. ab37106, Abcam, Cambridge, UK, 1:100 dilution), biotinylated secondary antibody, and avidin-biotinylated horseradish-peroxidase macromolecular complex (ABC kit, Vector Laboratories, Burlingame, CA). Peroxidase activity was visualized using diaminobenzidine as chromogen. Sections were counterstained with hematoxylin prior to mounting. The sections incubated in normal serum instead of the primary antibody served as negative controls (results not shown). For quantification of QA immunoreactive cells, from each rat, four sections were selected. Number of immunoreactive cells in six randomly selected fields in each section for each region (Cortex, CA1, CA3, dentate gyrus and thalamus) were counted at × 40 magnification in an Olympus microscope (BH-2) fitted with Nikon digital camera (Nikon Digital Sight DS-Fi1). NIS-elements D2.20 software was used for quantification of immunoreactive cells. At × 40 objective, each field was 56,575 μm^2^ in area. Mean number of immunoreactive cells/field in each region in each rat was calculated. Finally, mean ± SEM was calculated for each group.

### Statistical analysis

Data were expressed as Mean ± SD. Differences in the expression of various biochemical markers (by Western blot) and the levels of QA in the blood of VC and QA-exposed animals were analyzed by a Student’s *t* test for two independent samples with unequal variance. A *p* < 0.05 was considered statistically significant. For the spatial learning test, we averaged the escape latencies across the four trials for each rat. These means were then analyzed across the six sessions. A two-way repeated measures ANOVA was used for main effect (treatment comparisons) with sessions as the repeated measure and escape latency as the dependent variables. For the probe tests (spatial memory), group means were compared by Student’s *t* test for all parameters. Data were analyzed by SPSS version 23 for Windows (SPSS Inc., Chicago, IL, USA).

## Results

### Effects of lead exposure on quinolinic acid levels in the blood and brain

**Fig. 1:**
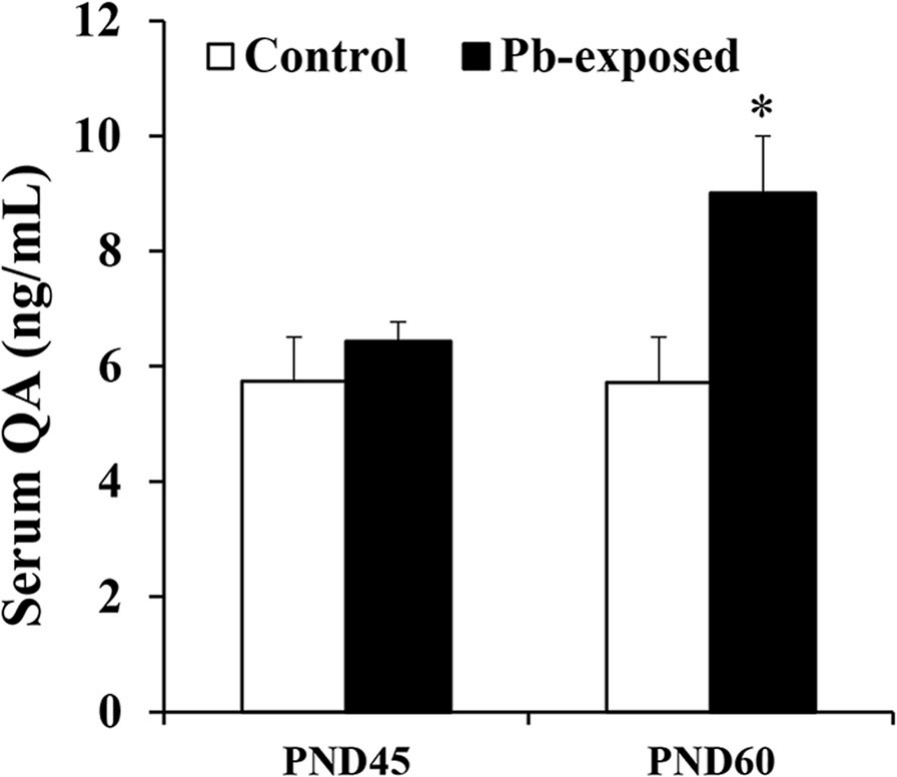
Serum quinolinic acid levels (ng/mL) in Pb-exposed Wistar rats. Data is mean ± SD (*n* = 6 in each group); the group means were compared with Student’s *t* test for two independent samples with unequal variance. Note significant increase in QA in serum at PND60 compared to control, but not at PND45, (*p < 0.05)

**Fig. 2:**
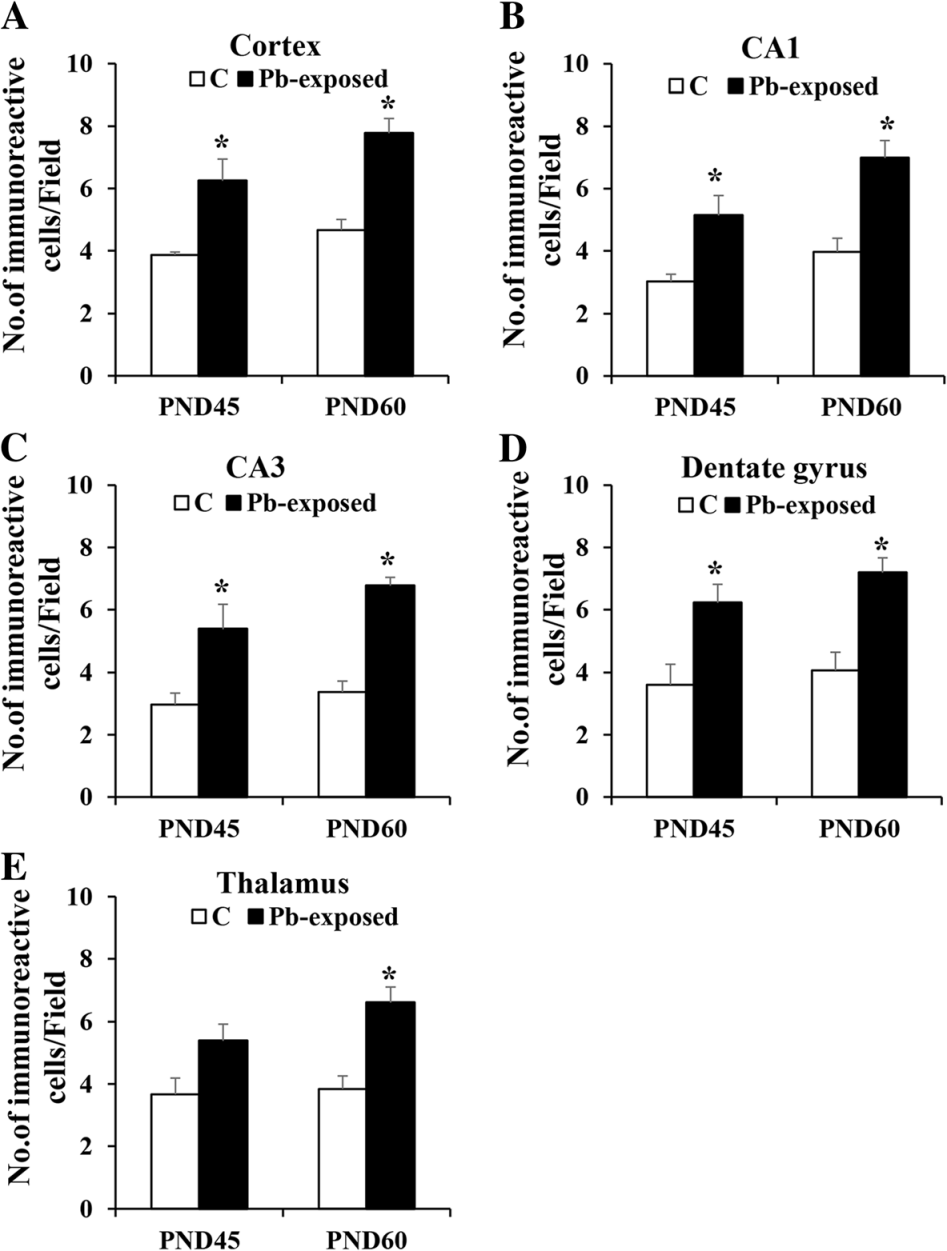
Number of quinolinic acid immunoreactive cells/field in cortex (**a**), CA1 (**b**), CA3 (**c**), dentate gyrus (**d**), and thalamus (**e**) regions in Pb-exposed and control groups at PND45 and PND60. Data were analyzed with one-way ANOVA with Bonferroni multiple comparisons test (*n* = 4 in all groups). Note Pb-exposed rats had significantly higher number of QA-immunoreactive cells in all regions studied at both PND45 and PND60 compared to control group except in thalamus at PND45 (* *p* < 0.05)

**Fig. 3:**
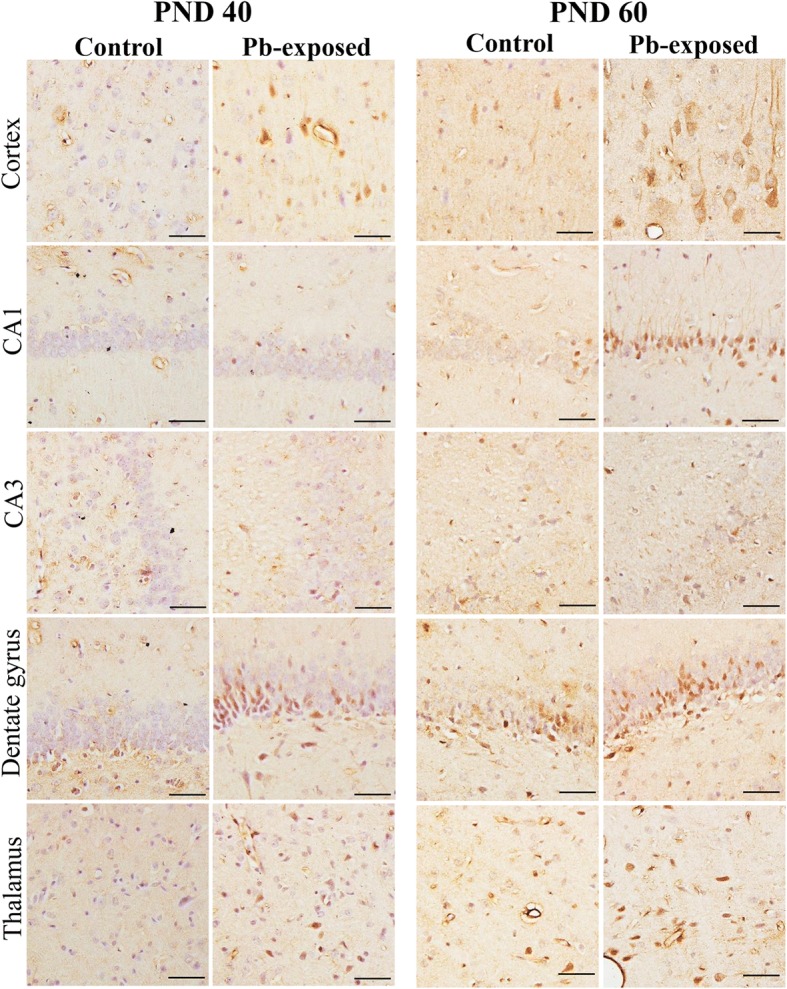
Photomicrographs of cortex, CA1, CA3, dentate gyrus, and thalamus regions in Control and Pb-exposed groups immunostained for QA at PND45 and PND60. Note higher number of immunoreactive cells in the Pb-exposed group in all regions. Scale bar = 40 μm

Rat pups exposed to 0.2% Pb-acetate from PND1 to PND21 or control pups were sacrificed at PND45 or PND60 after completion of spatial learning and memory tests in the MWM. QA was measured in serum samples by ELISA. As shown in Fig. 1, Pb exposure did not affect the level of QA in serum in the PND45 pups (5.74 ± 0.77 vs 6.43 ± 0.35 ng/ml in control and Pb-exposed groups, respectively). In the PND60 pups, Pb exposure significantly increased QA levels as compared to control group (5.72 ± 0.80 vs 9.01 ± 1.0; *p* < 0.05). In brain, the number of QA-containing neurons in Pb-exposed and control pups was compared by immunohistochemistry. QA-immunoreactive cells were counted in the cortex, thalamus, and three areas of the hippocampus (CA1, CA3, and dentate gyrus). Pb-exposed rats had significantly higher number of QA-immunoreactive cells in all regions studied at both PND45 (*p* < 0.05) and PND60 (*p* < 0.05) compared to the control group except in the thalamus at PND45 (Fig. 2). Representative photomicrographs of QA-immunoreactivity in control and Pb-exposed brain sections are shown in Fig. 3.

### Effects of Pb-exposure on spatial learning and memory

**Fig. 4:**
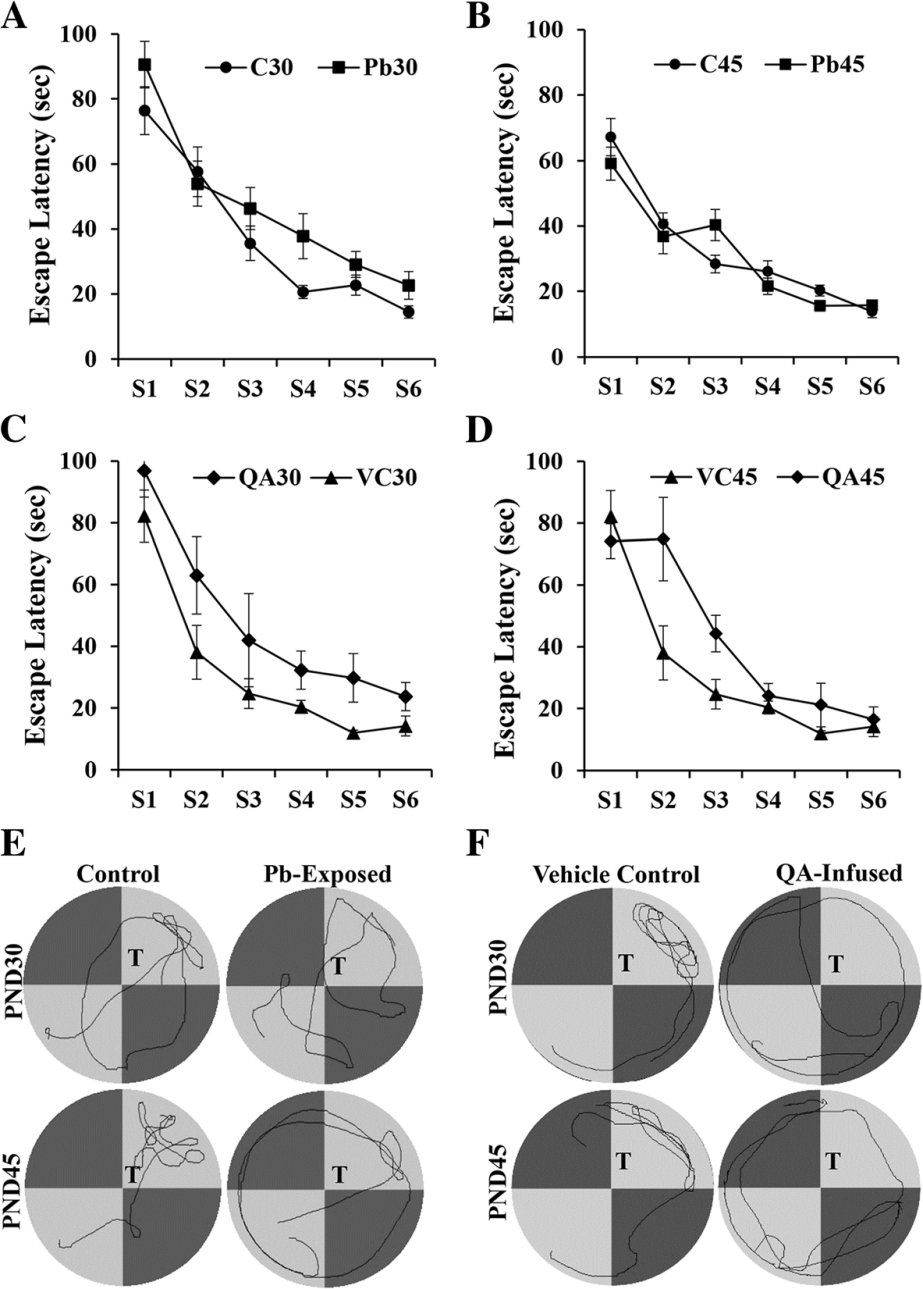
Escape latency of control and Pb-exposed rats (**a** and **b**), and vehicle control and QA-infused (**c** and **d**) in the Morris water maze: Learning sessions commenced at PND30 (**a** and **c**) and PND45 (**b** and **d**). **a**, **b** Note escape latency decreased from session to session both in control and Pb-exposed rats in PND30 and PND45 groups indicating that learning occurred. In the PND30 group, two-way repeated measures ANOVA revealed that the Pb-exposed groups learned significantly slower than the control group (*F* = 2.96; *p* = 0.034). In the PND45 group, the difference in the escape latencies across the sessions was not statistically significant (*F* = 2.12; *p* = 0.111). **c**, **d** Note escape latency decreased from session to session both in vehicle control and QA infused rat in PND30 and PND45 groups indicating that learning occurred. Two-way repeated measures ANOVA revealed that at PND30, the QA group learned significantly slower compared the vehicle control group (*F* = 3.52; *p* = 0.046). At PND45, the difference between VC and the QA groups was not statistically significant (*F* = 0.66; *p* = 0.527). **e**, **f** Representative video tracks of the probe test in the Morris water maze test of control and Pb-exposed (**e**) and vehicle control and QA-infused (**f**) rats. T, Target/platform quadrant

The effect of Pb-exposure on spatial learning was measured in MWM over six sessions starting at PND30 or PND45. During learning sessions, the escape latencies progressively became shorter in both groups (Pb-exposed and control) at both PND30 (Fig. 4a) and at PND45 (Fig. 4b), suggesting that learning occurred in both groups. In the PND30 group, repeated measures ANOVA revealed that the Pb-exposed groups learned significantly slower than the control group (*F* = 2.96; *p* = 0.034). However, in the PND45 group, the difference in the escape latencies across the sessions was not statistically significant (*F* = 2.12; *p* = 0.111). In the probe test conducted 2 days after the last learning session (STM), Pb-exposed rats in the PND30 groups spent less time (zone time), took longer time to enter (entry latency), and traveled less distance (distance) in the target/platform zone compared to control rats (Table 2), and the differences were significant for all the three parameters (*p* < 0.05). Among the PND45 rats, the difference in the zone time and entry latency was significantly different (*p* < 0.05), whereas, the distance was not significantly different. Overall, these results show that STM was impaired in the Pb-exposed puts in both PND30 and PND45 pups. Representative tracks of the probe test are shown in Fig. 4e. No effect was seen on LTM tested after 10 days of the learning sessions either in PND30 or in PND45 pups (data not shown).

**Table 2:** Effect of Pb exposure on short-term spatial memory (probe test) of Wistar rat pups at PND30 and PND45

	PND30		PND45	
	Control	Pb-exposed	Control	Pb-exposed
Zone time (s)	15.9 ± 0.84	10.5 ± 1.7*	18.6 ± 1.9	11.6 ± 1.1*
Entry latency (s)	8.6 ± 1.8	15.9 ± 2.7*	5.1 ± 0.8	8.4 ± 1.6*
Distance (cm)	345.0 ± 40.7	212.8 ± 33.6*	293.6 ± 53.4	313.0 ± 28.8
*N*	10	14	9	13

Data shown as mean **±** SEM. *Significantly different from respective control (*p* < 0.05) by Student’s *t* test for two independent samples with unequal variance

*Zone time****:*** Total amount of time (in sec) that the subject’s center of gravity spent in the target zone during the current trial. *Entry latency:* Time (in sec) before the subject’s center of gravity entered the target zone during the current trial. *Distance***:** distance traveled (cm) by the subject within the target zone

### Effects of intraventricular infusion of QA on spatial learning and memory

Rat pups were infused with 9 mM QA into the right lateral ventricle via osmotic minipumps from PND21 to PND27. VC group was similarly infused with saline. Pumps were removed from both groups on PND28, and the MWM was conducted at PND30 or at PND45. Similar to the Pb-exposed and control rats, learning occurred in both QA and VC groups, as the escape latencies progressively became shorter over the sessions at both PND30 (Fig. 4c) and at PND45 (Fig. 4d). Two-way repeated measure ANOVA revealed that at PND30, the QA group learned significantly slower compared to the vehicle control group (*F* = 3.52; *p* = 0.046). At PND45, the difference between VC and the QA groups was not statistically significant (*F* = 0.66; *p* = 0.527). The results of the probe test for STM are shown in Table 3. At PND30 and PND45, the QA-infused pups spent less time (zone time), took longer time to enter (entry latency), and traveled less distance (distance) in the target/platform zone compared to control rats; however, these differences were statistically significant only in the PND45 group (*p* < 0.05). Overall, these results show that QA infusion impaired STM compared to the VC groups, particularly in the PND45 rats. Representative tracks of the probe test are shown in Fig. 4f. Similar to the results of the Pb-exposure, QA infusion did not show any effect on LTM tested after 10 days of the learning sessions in either PND30 or in PND45 pups (data not shown).

**Table 3:** Effect of QA infusion on short-term spatial memory (probe test) of Wistar rat pups at PND30 and PND45

	PND30		PND45	
	Vehicle control	QA-infused	Vehicle control	QA-infused
Zone time (s)	9.7 ± 2.6	6.1 ± 1.9	18.6 ± 0.7	10.8 ± 1.9*
Entry latency (s)	9.7 ± 2.6	19.6 ± 4.7	5.5 ± 0.9	13.9 ± 0.9*
Distance (cm)	230.6 ± 74.6	154.6 ± 52.4	441.4 ± 43.3	233.5 ± 42.0*
*N*	7	10	8	11

Data shown as mean **±** SEM. *Significantly different from respective control (*p* < 0.05) by Student’s *t* test for two independent samples with unequal variance

*Zone time*, total amount of time (in sec) that the subject’s center of gravity spent in the target zone during the current trial; *Entry latency*, time (in sec) before the subject’s center of gravity entered the target zone during the current trial; *Distance*, distance traveled (cm) by the subject within the target zone

### Effects of intraventricular infusion of QA on the NMDA receptor subunits

**Fig. 5:**
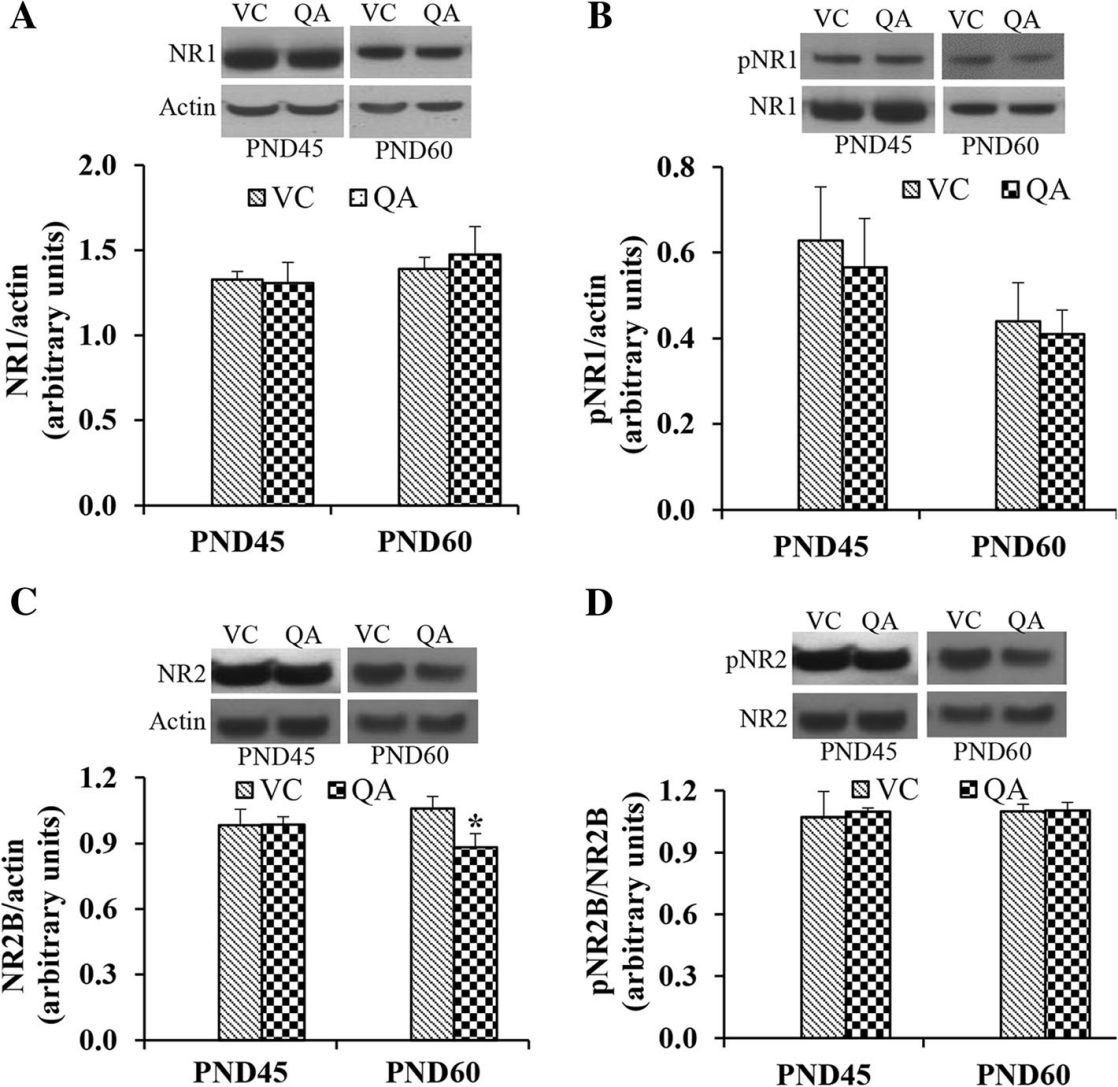
Expression of NR1 (**a**) and NR2B (**c**) subunits of NMDAR and phosphorylation of NR1 (**b**), and NR2B (**d**) in the control and QA-infused rats at PND45 and PND60. NMDAR subunit signal was normalized to actin signal. For pNR1 (at serine 897) and pNR2B (at serine 1303), the signal was normalized to the unphosphorylated protein signal. Representative blots are shown above each graph. Data presented as mean ± SD (*n* = 4); mean data were compared with Student’s *t* test. Note significantly decreased expression of NR2B at PND60 (*p < 0.05)

As QA is an NMDAR agonist, we investigated the changes in the expression of NR1 and NR2B subunits of NMDAR and their phosphorylation status at the end of learning and memory assessment (at PND45 and PND60). As shown in Fig. 5, QA-infusion did not affect the expression of NR1 (Fig. 5a) or its phosphorylation at serine 897 (Fig. 5b) either at PND45 or at PND60. At PND45, QA infusion did not affect the expression of NR2B, whereas at PND60, it significantly decreased the expression of this subunit (Fig. 5c). Similar to the NR1 phosphorylation, no effect of QA infusion on the phosphorylation of NR2B at serine 1303 was seen (Fig. 5d).

### Effect of intraventricular infusion of QA on signaling molecules, serine/threonine protein phosphatases and tau

**Fig. 6:**
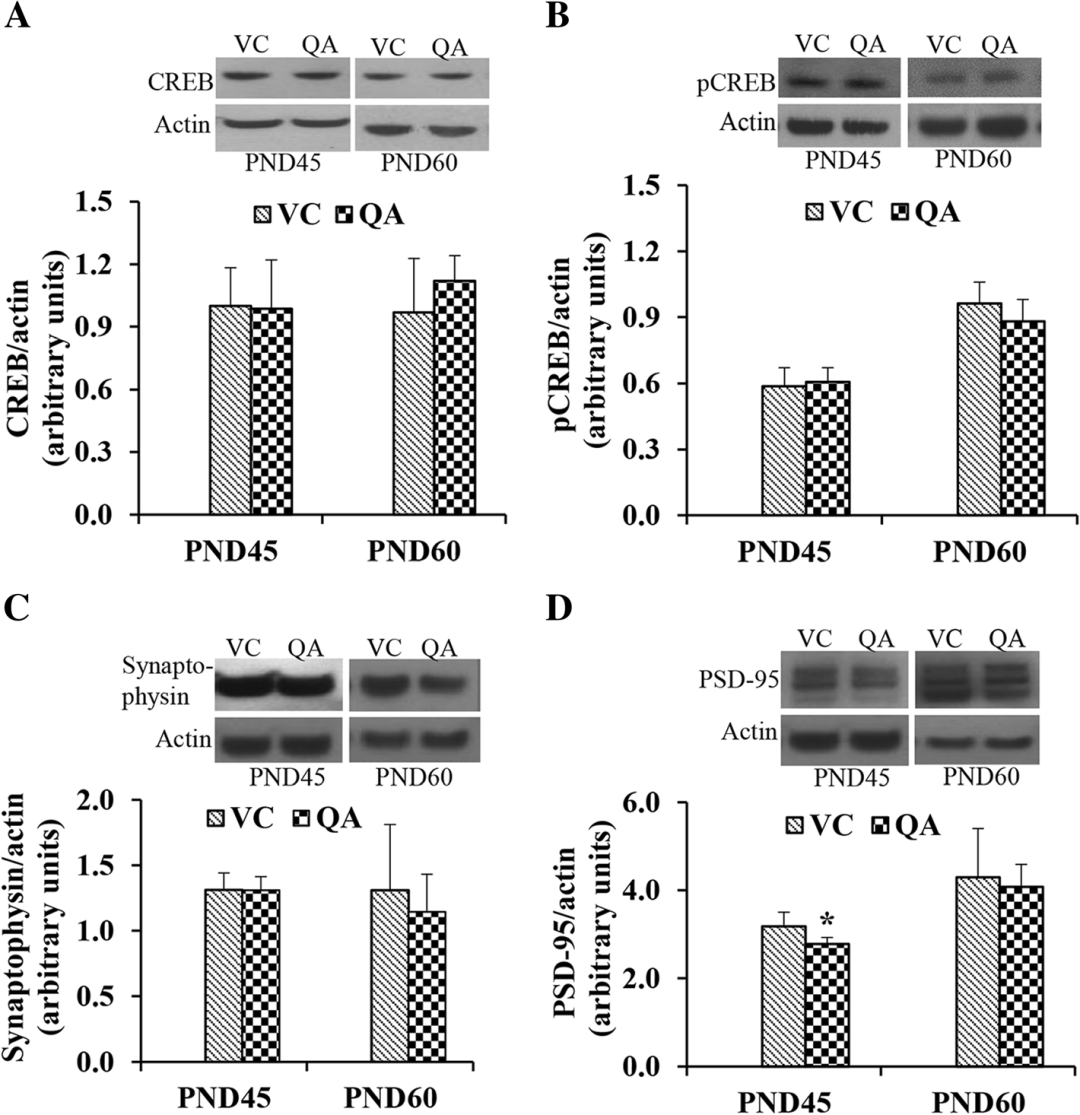
Expression of CREB (**a**), pCREB (**b**), synaptophysin (**c**), and PSD-45 (**d**) in the control and QA-infused rats at PND45 and PND60. All protein expressions were normalized to actin signal. Representative blots are shown above each graph. Data presented as mean ± SD (n = 4); mean data were compared with Student’s *t* test. Note significantly decreased expression of PSD-45 at PND45 (*p < 0.05)

**Fig. 7:**
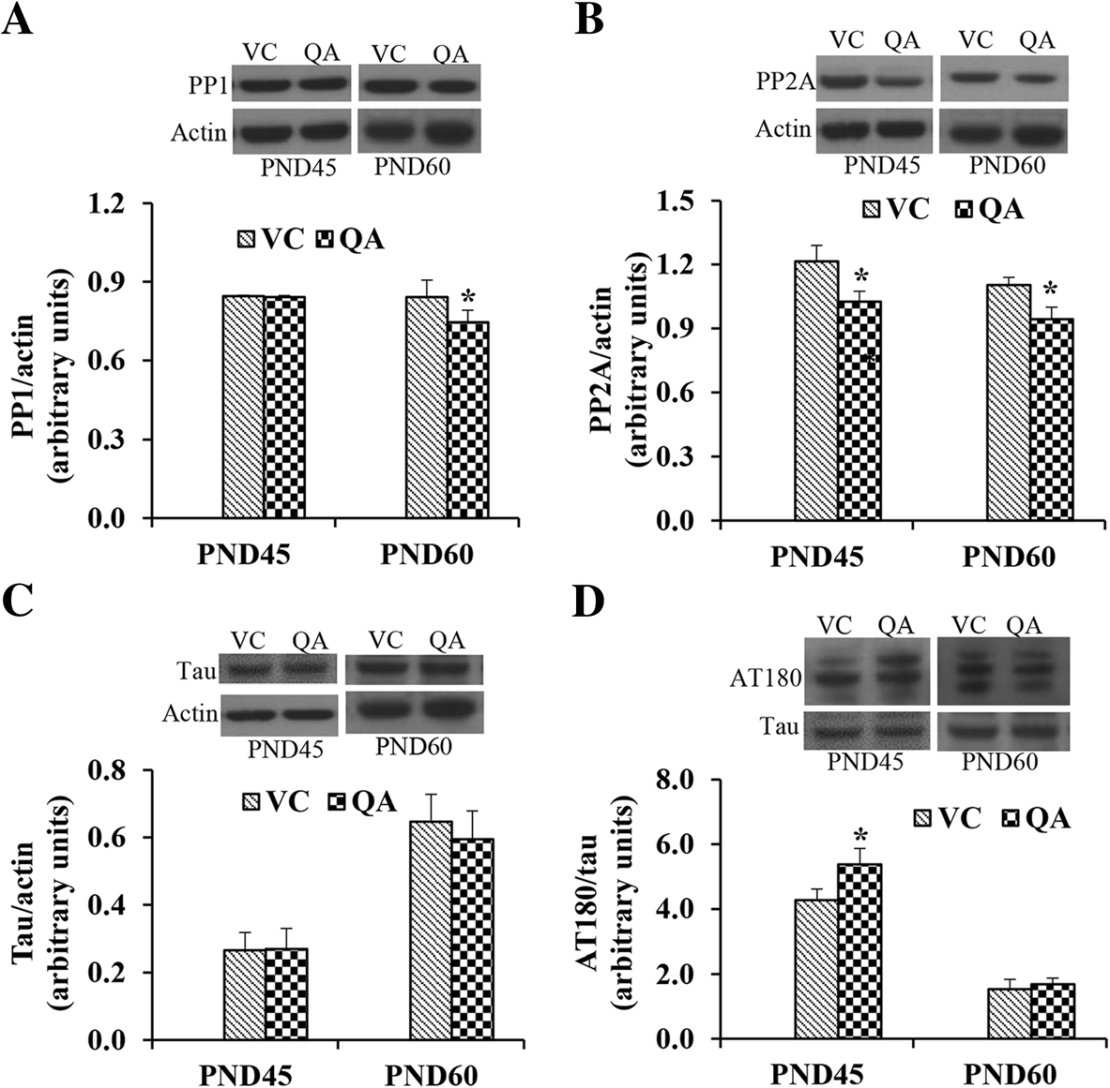
Expression of PP1 (**a**), PP2A (**b**), Tau (**c**), and phosphorylated tau at threonine 231 (**d**) in the control and QA-infused rats at PND45 and PND60. PP1A, PP2A, and Tau expression was normalized to actin signal; pTau at threonine 231 expression was normalized to Tau signal. Representative blots are shown above each graph. Data presented as mean ± SD (*n* = 4); mean data were compared with Student’s *t* test. Note significantly decreased expression of PP1 at PND60, decreased expression of PP2A at both PND45 and PND60, increased expression of AT180 at PND45 (*p < 0.05)

We also investigated the effect of QA infusion on various signaling molecules involved in learning and memory. These include CREB, CREB phosphorylated at serine 133 (pS^133-^CREB), synaptophysin, PSD-95, PP1, PP2A, tau and tau phosphorylated at AT-180 site; threonine 231 (pT^231^). QA-infusion did not show any effect on the expression of CREB (Fig. 6), pS^133^-CREB (Fig. 6b) or synaptophysin (Fig. 6c) either at PND45 or at PND60. The expression of PSD-95 was significantly decreased (*p* < 0.05) at PND45, but not at PND60 in the QA group compared to the VC group (Fig. 6d). The expression of PP1 was not affected at PND45, whereas it was significantly decreased at PND60 in the QA-infused pups (Fig. 7a). On the other hand, PP2A expression was significantly decreased at both PND45 and PND60 by QA-infusion (Fig. 7b). The expression of total tau was not affected by QA-infusion at either age (Fig. 7c), whereas its phosphorylation at threonine 231 was significantly increased at PND45 but not at PND60 (Fig. 7d).

## Discussion

In this study, we hypothesized that Pb-induced neurotoxicity involves, at least in part, increased levels of QA. Our results show that Pb exposure not only resulted in significant increase in the number of QA-immunoreactive neurons in the brain but also increased QA levels in serum. To our knowledge, this is the first study reporting increased QA production in response to Pb exposure. Pb, a pro-oxidant metal, may increase QA levels by microglial and astroglial activation. Microglial and astroglial activation in the brain, and in particular in the hippocampus, has been reported in Pb-exposed rats and mice. This microglial and astroglial activation was associated with increased expression of pro-inflammatory cytokines like IL-1β, IL-6, and TNF-α [37–43]. Similarly, in vitro Pb exposure of BV-2 mouse microglia resulted in increased expression of pro-inflammatory cytokines (TNF-α, IL-6, MCP-1) and pro-inflammatory enzyme COX-2 [44]. Thus, the generation of pro-inflammatory mediators and cytokines by Pb seems a logical mechanism for increased production of QA.

We then tested the effect of QA, infused directly into the ventricle, on learning and memory to investigate if Pb exposure and QA produce similar impairment of learning and memory. It is assumed that QA infused into the right ventricle will be circulated throughout the brain and will mimic increased levels of QA in the brain caused by Pb exposure. Our results show that similar to Pb-exposure, QA infusion resulted in significant impairment of learning at PND30 but not at PND45. This lack of effect of learning impairment in the PND45 rats may be explained by the clearance of Pb or QA from the brain over time. Pb exposure significantly impaired STM at both PND30 and PND45. QA infusion also impaired STM; however, the impairment was only statistically significant at PND45. The lack of statistical significance in memory impairment at PND30 could be explained by the small number of rats (7–8 in the QA group) in these experiments. The lack of effect of Pb or QA on learning at PND45 but significant impairment of STM at this age suggest that effects of both Pb and QA on STM are long-lasting compared to their effect on learning. It may also suggest that the effects of both Pb and QA on learning process and memory recall are different. The prolonged effect on memory recall, but not learning, may be due to the adverse effects of Pb and QA on molecules, receptors and specific structural components (e.g. synapses, dendritic field, dendritic spines) concerned with memory recall. Alternatively, Pb and QA may affect the memory consolidation process itself. Overall, QA infusion mimics the effects of exposure of Pb through dams’ drinking water, in producing similar effects on learning and STM, supporting our hypothesis of QA involvement in Pb-induced neurotoxicity.

A similar QA infusion protocol in adult Sprague-Dawley rats resulted in learning impairment and short-term working memory deficits [32]. The working memory deficits caused by QA infusion in this study lasted for up to 3 weeks after the termination of infusion, and these results conform to our findings. Cognitive deficits caused by QA infusion in the brain of Sprague-Dawley rats were prevented by simultaneous subcutaneous infusion of memantine, an NMDA receptor antagonist [33], suggesting that QA impaired learning and memory through NMDAR activation. In other studies, a single injection of QA into the striatum of rats also caused significant memory impairment [31, 45].

The effects of Pb on NMDAR have been extensively studied, and it has been reported that Pb affects NMDAR function by modulating the expression and the subunit composition of the receptor (Reviewed by Rahman [1]). Furthermore, these effects seem to be brain region-specific. For example, Pb exposure decreased the expression of NR1 and NR2B subunits in hippocampal neurons, whereas in cortical neurons, no effect was seen on the expression of NR1 and the expression of NR2B was significantly increased [46, 47]. We investigated if QA infusion in the brain would produce similar effects on the NR1 and NR2B subunit expression. We did not find any changes in the protein levels of NR1 at either PND45 or PND60. On the other hand, the expression of NR2B was not affected at PND45, but was significantly decreased by QA infusion in the brain lysate of PND60 rats. The decrease in NR2B content in the brain may be due to the normal ontogenic changes in the brain. During early postnatal stage, the expression of NR2B containing NMDAR predominates. A developmental switch occurs later, which is accompanied by an increase in the NR2A-containing receptors and a subsequent decrease in NR2B containing receptors [41]. NR2B-containing NMDAR are more abundant in the brain as these are expressed both in synapses and outside the synapses, compared to NR2A containing NMDAR, which are predominantly located in synapses [48]. An increase in the synaptic NR2B-containing receptor without any change in the overall cellular level of NR2B in response to Pb exposure has been reported [47, 49]. In the Zebrafish embryo, Pb decreased the expression of NR1 and had no effect on the expression of NR2B [50]. Further research is needed to elucidate the temporal and brain-region-specific changes in the expression of these subunits in response to QA administration.

CREB is a transcription factor for many NMDAR activity-dependent immediate early genes which play an essential role in learning and memory [51, 52]. CREB plays an essential role in the propagation of signal to the nucleus by linking NMDAR activation to the expression of genes necessary for synaptic plasticity [53]. CREB is phosphorylated in response to NMDAR activation and induction of long-term potentiation (LTP). CREB phosphorylation at S^133^ facilitates the recruitment of CREB-binding proteins and the assembly of transcriptionally active complex at the start site of CRE containing genes [54]. We looked at the effect of QA infusion on the expression of CREB and its phosphorylation at S^133^. No effect was seen on the total level of CREB or its phosphorylation at S^133^ in response to QA infusion. Similar to this, no effect of Pb exposure was seen on the total level of CREB, but in contrast to the effect of QA, Pb exposure decreased the phosphorylation of CREB [49, 55].

Synaptophysin is a major protein of synaptic vesicles and plays an important role in synaptic transmission, stabilization, and plasticity [56, 57]. It is involved in neurotransmitter release and synaptic vesicle cycle and thus is used as marker of synaptic terminals [58, 59]. A decrease in the expression of synaptophysin in rats and mice exposed to Pb has been reported, and this decrease in synaptophysin level was suggested to be a potential mechanism of Pb neurotoxicity [47, 60–62]. On the other hand, Gassowska et al. [63] reported increased synaptophysin expression in the cerebellum of rat pups perinatally exposed to Pb. In our study, QA infusion did not affect the level of synaptophysin in the whole brain lysate of rats. Similar to our results, intra-striatal injection of QA into rat brain showed no change in the expression of synaptophysin [64]. Thus, the effect of QA on synaptophysin appears to be different from the effect of Pb exposure.

At the postsynaptic site, neurotransmitter receptors, signaling enzymes, and cytoskeletal proteins are clustered along with scaffolding proteins in a structure called postsynaptic density [65]. PSD-95 is an important component of postsynaptic density which is highly expressed in glutamatergic synapses along with NMDAR [63]. PSD-95 increases the number and the size of dendritic spines and contributes to synaptic stabilization and plasticity [66–68]. Reduction of PSD-95 in neurodegenerative diseases of the brain, such as Alzheimer’s disease and Parkinson’s disease, has been reported [69–71]. In rat pups, perinatal Pb exposure decreased the expression of PSD-95 in the forebrain, cortex, and cerebellum, but increased its expression in the hippocampus [63]. In mice, developmental Pb exposure also resulted in decreased mRNA and protein levels of PSD-95 at PND40 [62]. We observed a significant decrease in PSD-95 level in the brain in response to QA infusion at PND45 but not at PND60. The lack of effect at PND60 is likely due to the clearance of QA and its effects from the brain with time. In a previous study, prenatal inhibition of the KP by Ro61-8048 administration into the dams, which inhibits the enzyme kynurenine-3-monoxygenase and shifts the pathway in favor of kynurenic acid, resulted in increased expression of PSD-95 in the PND21 rat pups [72]. These results are parallel to our findings. As PSD-95 is a marker for synapses, we speculate a decrease in the number of synapses in the brain of QA-infused rats. We have previously shown a decrease in the number of synapses in the hippocampus of rat pups developmentally exposed to Pb [30]. Overall, the effect of QA infusion on PSD-95 expression is similar to the effect of Pb exposure on PSD-95. The effect of QA infusion on the number of synapses, particularly in the hippocampus, needs to be investigated.

Whereas, LTM involves protein synthesis, growth, and formation of new synapses [73–75], STM is regulated by covalent modification of proteins in the presynaptic or postsynaptic structures [76–78]. Reversible protein phosphorylation is one of the major covalent modifications involved in this process and is regulated by the balance between protein kinases and protein phosphatases. Of the protein phosphatases, serine/threonine protein phosphatases PP1 and PP2A are the major phosphatases in the brain, accounting for over 90% of the total mammalian brain protein phosphatase activity [78]. Any alteration in the activity of these two phosphatases may significantly affect the phosphorylation state of proteins. A decrease in the activities of these phosphatases has been implicated in Alzheimer’s disease. [79]. On the other hand, overactivation of these phosphatases are also reported to be involved in learning and memory impairment (reviewed by Rahman et al. [1, 30]). PP1 and PP2A are located in physical proximity to NMDAR [80]. Following excitatory neurotransmission and Ca^2+^ influx, NMDAR are phosphorylated and then rapidly dephosphorylated. This reversible phosphorylation controls synaptic strength, memory formation, and storage by the induction of LTP or long-term depression (LTD) [81]. Phosphorylated NMDAR have enhanced channel openings, and the consequent increase in Ca^2+^ influx is implicated in the induction of LTP [82–85]. Dephosphorylation of the NMDAR on the other hand is implicated in the induction of LTD [86, 87]. Stimulation of NMDAR activates PP1 and PP2A [88]. Downstream of the NMDAR, these phosphatases dephosphorylate CREB [89] and thereby reduce CREB-mediated gene expression [90–93].

Pb is known to dysregulate serine/threonine protein phosphatases in the brain. We have previously reported that early postnatal Pb exposure resulted in increased PP1 but decreased PP2A levels in the brain of rat pups at PND21 [34]. We also reported a decrease in PP2A levels in the hippocampus of rats pups at PND30 [28]. We therefore investigated the effect of QA infusion on the levels of these phosphatases in the brain. PP1 expression was decreased only in the PND60 rats, whereas the expression of PP2A was decreased at both PND45 and PND60. Parallel to these results, we have previously reported a decrease in total brain phosphatase activity and in PP2A activity by QA in a dose-dependent manner in cultured human neurons. We also showed a decrease in the expression of both PP1 and PP2A in a dose-dependent manner [22]. An increase in the phosphorylation level of low molecular weight neurofilament subunit in neurons and the glial fibrillary acidic protein in astrocytes, which is consistent with decreased phosphatase activity, has been reported in response to a single injection of QA in the brain [25].

Tau is a microtubule associated protein which is required for the maintenance of intact microtubule structure. Disruption of microtubules has been shown to be associated with memory loss and neuronal death [94]. Abnormally hyperphosphorylated tau has not only less affinity for binding with microtubules, but also sequesters normal tau and other microtubule-associated proteins and causes disassembly of microtubules [95]. Tau hyperphosphorylation and its subsequent accumulation as paired helical filaments are implicated in neurodegenerative diseases and learning and memory impairment. Pb causes hyperphosphorylation of tau both in vitro [96] and in vivo in Pb-exposed animals [34]. In this study, we showed that QA infusion resulted in the phosphorylation of tau at T^231^, without affecting total tau. We have previously reported a dose-dependent increase in the phosphorylation of tau at several residues including T^231^ in cultured human neurons exposed to QA [20]. Tau at this site was also phosphorylated by Pb in our previous studies [34, 96]. Hyperphosphorylation of tau at T^231^ results in tau self-assembly [97]. QA-induced NMDAR activation appears to be the mechanism of this increased tau phosphorylation, as both glutamate and NMDA increased tau phosphorylation at sites similar to QA. Furthermore, MK-801, an NMDAR antagonist, inhibited tau phosphorylation at AT-180 site [22, 98]. These results clearly indicate that both Pb and QA have similar effects on tau phosphorylation, further supporting our hypothesis of QA involvement in Pb-induced neurotoxicity.

## Conclusion

**Fig. 8:**
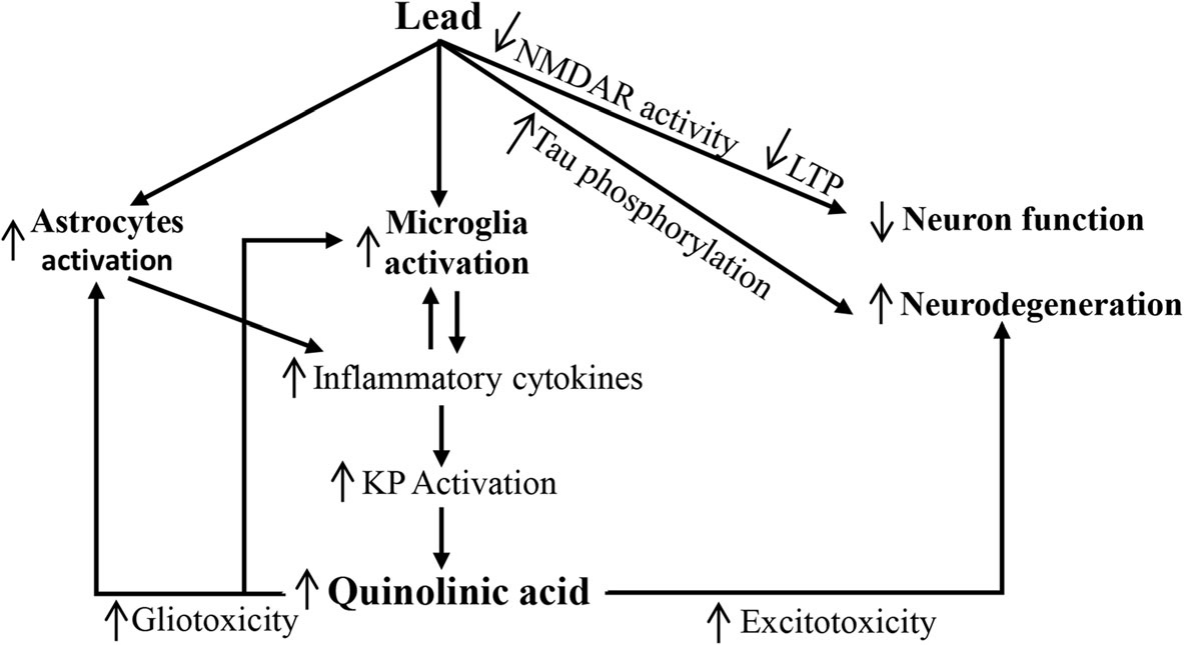
Schematic representation of probable role of quinolinic acid in lead-neurotoxicity. Pb accumulation in the brain results in decreased NMDAR activity, which results in decreased LTP leading to impaired learning and memory (reviewed by Rahman, 2013 [1]). In addition, Pb induces astroglyosis and microglyosis [38]. Activated astrocytes and microglia produce inflammatory cytokines, which activates the KP, converting tryptophan into QA [11]. High levels of QA are toxic to astrocytes, microglia, and neurons [12, 14]. QA is an NMDAR agonist and causes excitotoxicity in neurons [10]. In addition, it increases synaptic glutamate levels, further increasing excitotoxicity [35]. This increased excitotoxicity in neurons, together with tau hyperphosphorylation [22, 98], eventually results in neurodegeneration. Up arrows indicated upregulation/increase; down arrows indicate downregulation/decrease

In this study, two experimental paradigms were used. In the first experiment, exposure of rat pups (via their dams’ drinking water) during early postnatal period resulted in increased QA levels in the blood and the number of QA-immunoreactive cells in the brain, and impaired learning and short-term memory. In the second experiment, direct intraventricular infusion of QA during early postnatal period significantly impaired learning and short-term memory, similar to the first experiment (Pb exposure). Intraventricular infusion of QA decreased the levels of PSD-95, PP1, and PP2A, and increased tau phosphorylation. All these effects are parallel to the reported effect of Pb exposure. Putting together the results of these experiments, we propose increased QA production as a novel mechanism of Pb-induced neurotoxicity. Our proposed model is depicted in Fig. 8. Further research is needed to elucidate the mechanism by which Pb exposure results in increased QA production. In addition, the potential beneficial effects of NMDAR antagonists, particularly memantine, needs to be investigated in Pb-induced learning and memory impairment.

## Abbreviations

IDO-1: Indoleamine-2,3-dioxygenase-I
KP: Kynurenine pathway
LTM: Long-term memory
MWM: Morris water maze
NMDA: *N*-Methyl-d-aspartic acid
NMDAR: *N*-Methyl-d-aspartic acid receptor
Pb: Lead
PND: Postnatal day
QA: Quinolinic acid
STM: Short-term memory
VC: Vehicle control
